# 1014. Patient perspectives on pharmacy experiences for antiretroviral refills

**DOI:** 10.1093/ofid/ofac492.855

**Published:** 2022-12-15

**Authors:** Josh Havens, Yadi Liu, Renae Furl

**Affiliations:** University of Nebraska Medical Center, Omaha, Nebraska; University of Nebraska Medical Center, Omaha, Nebraska; University of Nebraska Medical Center, Omaha, Nebraska

## Abstract

**Background:**

Control and prevention of HIV infection require sustained adherence to antiretroviral therapy (ART) for patients with HIV (PWH) or those on pre-exposure prophylaxis (PrEP). ART is increasingly designated by payers as a specialty medication and often mandates specialty mail-order pharmacy for refills. Little is known about patient perspectives of pharmacy choice preferences for ART.

**Methods:**

A 20-question survey was distributed to patients (PWH and PrEP) of the Specialty Care Center in Omaha, NE. Participants >19 years, retained in care for >6 months, and experience with ART/PrEP refills at both local and mail-order pharmacies were considered for study participation. The survey was designed in 3 parts: perspectives on both pharmacy settings, ranking of pharmacy attributes, and pharmacy preference. Descriptive analyses were performed for all survey questions.

**Results:**

Surveys were completed by 61 participants (N=156; 39.1%). Median age was 52 years, 85% White, 13% Black, 5% Latinx, and 80% commercially insured. Forty-eight participants (79%) reported insurance mandates to use a specialty mail-order pharmacy with 28 (58%) believing the mandate impacted their medical care. Forty-one participants (67%) preferred refilling prescriptions through a local pharmacy. Participants ranked “ease of refilling” as the most important attribute (Figure 1) and favored local pharmacies vs mail-order pharmacies for all pharmacy attributes (Figure 2).

**Figure:**
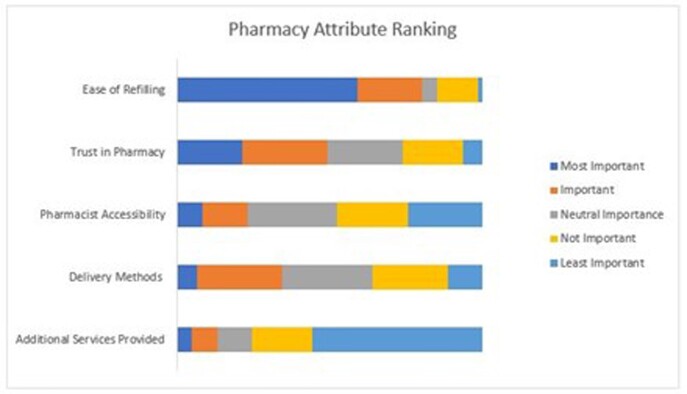

**Figure:**
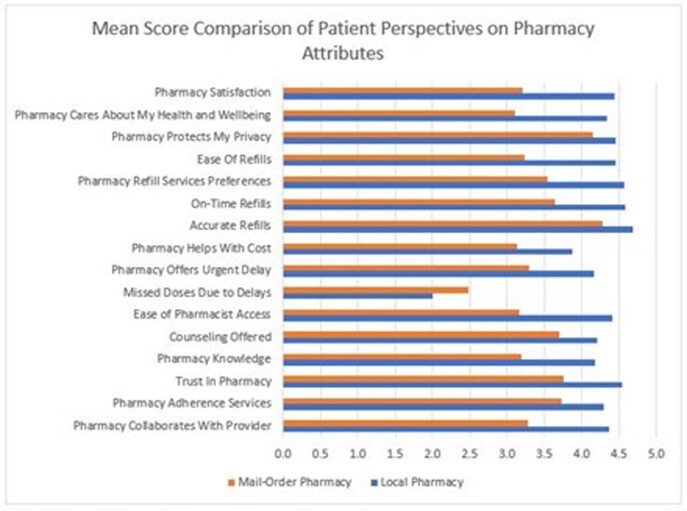

Scoring scale, 1=strongly disagree to 5=strongly agree.

**Conclusion:**

Midwest PWH and PrEP patients generally preferred local pharmacies to refill their antiretroviral therapies versus mail-order pharmacies for nearly all pharmacy attributes. Further, many participants felt specialty pharmacy mandates negatively impacted their medical care. Insurance payers should consider removing restrictive specialty pharmacy mandates for ART/PrEP prescriptions.

**Disclosures:**

**All Authors**: No reported disclosures.

